# Comparative Evaluation of Chemically Synthesized and Biosynthesized 25-Hydroxyvitamin D_3_ Supplementation in Sows During Late Gestation and Lactation

**DOI:** 10.3390/ani16172674

**Published:** 2026-08-26

**Authors:** Bangxin Xue, Fengju Liu, Jiale Bao, Shengjie Shi, Shuang Cai, Xiangzhou Zeng, Xinyu Wang, Zhekun Zhu, Guiyan Chu, Xiangfang Zeng, Shimin Liu, Peng Qin, Chuanjiang Cai, Zhenhua Xue

**Affiliations:** 1 State Key Laboratory of Animal Nutrition and Feeding, Ministry of Agriculture and Rural Affairs Feed Industry Centre, National Feed Engineering Technology Research Center, China Agricultural University, No. 2 Yuanmingyuan West Road, Beijing 100193, China; bs20243040485@cau.edu.cn (B.X.);; 2 Beijing Hilink Biotechnology Co., Ltd., Beijing 102206, China; liu19861205@163.com; 3 College of Animal Science and Technology, Northwest A&F University, Yangling 712100, China; 4 UWA School of Agriculture and Environment, University of Western Australia, Crawley, WA 6009, Australia; 5 Key Ingredients Biotechnology (Yichang) Co., Ltd., Yichang 443000, China; 6 The Municipal Animal Husbandry General Station of Beijing, A15 Beiyuan Road, Chaoyang District, Beijing 100107, China

**Keywords:** 25-hydroxyvitamin D_3_, vitamin D_3_, sow reproductive performance, piglet growth performance, skeletal development

## Abstract

Vitamin D and its metabolite 25-hydroxyvitamin D_3_ (25-OH-D_3_) are central to calcium homeostasis, maternal skeletal integrity, and sow reproductive efficiency. This study compared vitamin D_3_ with an equivalent supranutritional dose of chemically or microbially produced 25-OH-D_3_ from day 90 of gestation to weaning. A total of 100 Large White × Landrace sows were assigned to five treatments (*n* = 20): a basal control (25 µg/kg vitamin D_3_), a VD_3_ group (basal + 50 µg/kg vitamin D_3_), and three 25-OH-D_3_ groups (basal + 50 µg/kg 25-OH-D_3_; QV1–QV3). Reproductive traits, colostrum composition, serum vitamin D-related indices, antioxidant and cytokine profiles, pre-weaning piglet growth, femoral mineral concentration, intestinal morphology, and barrier-related gene expression were assessed. Compared with the control, QV2 increased litter weight gain from birth to day 14 by 9.8%, piglet serum 25-OH-D_3_ at weaning by 33.2%, and femoral calcium concentration by 14.4% (*p* < 0.05); litter and average piglet weights at weaning were unchanged. Maternal 25-OH-D_3_ supplementation also altered the overall redox profile and several circulating inflammatory cytokines, increased jejunal and ileal villus height in the QV2 and QV3 groups, and upregulated selected tight-junction-related genes, including occludin and ZO-1, in a source- and intestinal-segment-dependent manner (*p* < 0.05). Responses were source-dependent, and QV2 showed the strongest effects for selected endpoints; the data do not support class-wide superiority of biosynthesized preparations.

## Simple Summary

We compared chemically synthesized and microbially biosynthesized 25-hydroxyvitamin D_3_ at similar dietary inclusion levels from late gestation to weaning in sows. Maternal supplementation with 25-hydroxyvitamin D_3_ increased vitamin D status in sows and piglets and produced source-dependent changes in downstream vitamin D metabolites, antioxidant status, inflammatory markers, bone mineralization, and intestinal development. Among the three preparations, the yeast-derived QV2 treatment showed the strongest responses for selected outcomes, including greater litter weight gain from birth to day 14, higher colostrum 25-hydroxyvitamin D_3_ concentration, and increased femoral calcium concentration in weaned piglets. QV2 and QV3 also increased jejunal and ileal villus height. However, treatment rankings were not consistent across all measured endpoints, and litter and average piglet weights at weaning were not significantly affected. Therefore, these findings indicate product-specific responses and do not demonstrate general superiority of biosynthesized 25-hydroxyvitamin D_3_ preparations over chemically synthesized products.

## 1. Introduction

Sow reproductive efficiency is pivotal for herd productivity and is closely linked to maternal skeletal health, a key determinant of sow longevity [1]. During late gestation and lactation, sows must transfer large amounts of calcium to support rapid fetal growth and milk production, which inevitably increases mobilization of Ca from the maternal skeleton and predisposes sows to bone loss [2,3]. Importantly, evidence suggests that Ca supplementation alone is often insufficient to prevent this excessive skeletal mobilization in late gestation and lactation [3,4,5], indicating that additional nutritional or endocrine regulators of Ca homeostasis are required.

Vitamin D plays an important role in maintaining calcium homeostasis for bone mineralization and skeletal muscle development [6,7]. Dietary vitamin D_3_ undergoes sequential hydroxylation in the liver and kidneys to generate 1,25-(OH)_2_-D_3_, which regulates calcium balance by enhancing intestinal absorption and renal reabsorption and, when necessary, mobilizing skeletal calcium [8,9,10]. By contrast, 25-hydroxyvitamin D_3_ (25-OH-D_3_) bypasses hepatic hydroxylation and is more readily available for subsequent activation [11,12]. In pigs, maternal 25-OH-D_3_ supplementation has been reported to influence sow reproductive performance and colostrum composition and to support intestinal calcium absorption and bone properties in sow–piglet pairs [13,14]. Studies involving direct dietary supplementation of weaned piglets have associated 25-OH-D_3_ with bone quality, antioxidant and immune indices, intestinal morphology, and tight-junction-related gene expression [15,16]. Thus, the reproductive and colostrum evidence derives from maternal sow studies, whereas much of the intestinal and immune evidence derives from direct supplementation studies in weaned piglets. Direct head-to-head comparisons among 25-OH-D_3_ preparations produced by different chemical or microbial routes remain scarce, which provides the rationale and novelty for the present study.

Accordingly, we hypothesized that 25-OH-D_3_ preparations manufactured through different routes could produce source-dependent responses because differences in formulation, purity, stereochemical composition, stability, or recovery in feed may alter effective bioavailability even when the nominal supplemental dose is identical. The comparison was intended to evaluate biological responses among the available preparations rather than to attribute any observed difference solely to chemical or microbial origin. QV1 was commercially available, and chemically synthesized 25-OH-D_3_ was used as the reference material. QV2 and QV3 were biosynthesized 25-OH-D_3_ preparations produced using Saccharomyces cerevisiae and Bacillus subtilis as production strains, respectively. These strains were selected because they were the production hosts of the available candidate preparations and represented yeast- and bacterial-based biosynthetic platforms. Their inclusion was exploratory and was not based on prior evidence that one production strain would be biologically superior to the other in sows. The objective was to compare the three preparations during late gestation and lactation with respect to reproductive performance, maternal and offspring vitamin D-related indices, oxidative and inflammatory markers, piglet growth, femoral mineral concentrations, intestinal morphology, and barrier-related gene expression.

## 2. Materials and Methods

### 2.1. Animals and Experimental Design

This experiment was conducted at Saneng Pig Farm (Shanxi, China) and approved by the Animal Care and Use Committee of China Agricultural University (AW01705202-1-3, approval date: 10 July 2025). A total of 100 Large White × Landrace sows (parity: 4.25 ± 0.88; P2 backfat thickness: 17.50 ± 1.54 mm) were blocked by parity and P2 backfat thickness and then randomly assigned within each block to one of five dietary treatments (*n* = 20 per treatment): CON (basal diet containing 25 µg/kg vitamin D_3_), VD_3_ (CON plus 50 µg/kg vitamin D_3_), and QV1–QV3 (CON plus 50 µg/kg 25-hydroxyvitamin D_3_ from three sources). Diets were formulated to meet NRC [17] requirements for late gestation and lactation. The feeding period extended from day 90 of gestation to weaning (day 28 of lactation). Sows were individually housed with ad libitum access to water and received feed three times daily (08:00, 14:00, and 20:00).

### 2.2. Dietary Composition

Gestation and lactation diets were corn–soybean meal-based and supplemented with a vitamin–mineral premix (1%) and phytase. The premix supplied 25 µg/kg (1000 IU/kg) vitamin D_3_, with additional vitamin D_3_ or 25-OH-D_3_ provided according to treatment. Because mineral metabolism and skeletal outcomes were central to the study, the basal gestation and lactation diets contained 0.67% and 0.88% calcium, 0.68% and 0.59% total phosphorus, and 0.26% and 0.38% available phosphorus, respectively (Table 1).

#### Supplement Sources and Diet Verification

QV1 was a chemically synthesized 25-OH-D_3_ used as the reference material. QV2 and QV3 were biosynthesized 25-OH-D_3_ preparations produced using Saccharomyces cerevisiae and Bacillus subtilis as production strains, respectively. QV1, QV2, and QV3 were formulated premixes with a nominal 25-OH-D_3_ content of 0.05% (*w*/*w*), with modified starch used as the carrier. The analyzed 25-OH-D_3_ contents of the QV1, QV2, and QV3 premixes were 0.055%, 0.054%, and 0.056% (*w*/*w*), respectively, as determined according to the Chinese agricultural industry standard NY/T 3879-2021 [18], Determination of 25-Hydroxyvitamin D_3_ in Feeds. The premixes were added to the diets at 100 mg/kg, providing 50 μg/kg 25-OH-D_3_. The CON and VD_3_ diets received the same amount of modified starch carrier. Because the final dietary concentration was below the detection limit of the available method, dietary 25-OH-D_3_ concentrations are reported as formulated values.

### 2.3. Sample Collection

Reproductive traits (total born, born alive, stillborns, mummified fetuses, piglets weaned, and farrowing duration) were recorded. Sow body weight and P2 backfat were measured at farrowing and weaning to calculate backfat loss; feed allowance during gestation and daily intake during lactation were recorded. Piglets were weighed at birth, day 14, and weaning, and litter weight gain was calculated. Six sows per treatment were randomly selected for blood and colostrum sampling. Sow blood samples were collected on gestation day 90 before the initiation of the experimental diets, at farrowing, and at weaning, and colostrum samples were collected at farrowing (*n* = 6 per treatment). At weaning, one clinically healthy piglet whose body weight was closest to the mean body weight of its litter was selected from each of six sampled litters per treatment and euthanized for collection of serum, liver, duodenum, jejunum, ileum, femur, and tibia. Piglet sex was not controlled during selection. The subset size was specified before sampling based on assay and terminal-tissue capacity while retaining six independent sows or litters per treatment. When cross-fostering was required, piglets were transferred only among sows in the same treatment, and litter performance outcomes were calculated using the actual post-fostering litter composition.

### 2.4. Vitamin D Metabolites, Biochemical Indices, Antioxidants, Cytokines, and Immunoglobulins

Serum and colostrum 25-OH-D_3_ were measured using a competitive ELISA kit (HB402-SH), and ALP using an assay kit (L-726-SH) (Shanghai Hengyuan Biotechnology Co., Shanghai, China). Concentrations of 1,25-dihydroxyvitamin D_3_ [1,25-(OH)_2_-D_3_], 24,25-dihydroxyvitamin D_3_ [24,25-(OH)_2_-D_3_], and vitamin D-binding protein (DBP) were quantified using commercial ELISA kits (HY028-NC, HY094-SH, and HY350-Pg, respectively). Serum calcium was measured by a GBHA-based micro-colorimetric method (520 nm), and serum phosphorus by a phosphomolybdate microplate method (660 nm). Antioxidant indices were determined using porcine ELISA kits for SOD (HB326X-Pg), GSH-Px (HB463X-Pg), CAT (HB251X-Pg), and MDA (HB443-Pg). Cytokines (IL-1β, HB354-Pg; IL-6, HB347-Pg; IL-10, HB359-Pg; TNF-α, HB015-Pg; IFN-γ, HB363-Pg) and colostrum IgG (HS173-Pg) and IgM (HS170-Pg) were quantified by ELISA. All ELISA kits were purchased from Shanghai Hengyuan Biotechnology Co., Ltd. (Shanghai, China) and were used according to the manufacturer’s instructions. The assay ranges and analytical sensitivities were 0.3–20 μg/L and 0.1 μg/L for 25-OH-D_3_, 3.2–200 pg/mL and 3 pg/mL for 1,25-(OH)_2_-D_3_, 0.32–20 ng/mL and 0.17 ng/mL for 24,25-(OH)_2_-D_3_, and 15.6–1000 μg/mL and 9.8 μg/mL for DBP, 1–45 ng/L and 0.25 ng/L for IL-1β, 50–1000 ng/L and 12.5 ng/L for IL-6, 8–180 ng/L and 2 ng/L for IL-10, 0.3–8 nmol/L and 0.075 nmol/L for MDA, 40–2000 U/L and 10 U/L for SOD, 5–165 IU/L and 1.25 IU/L for GSH-Px, 35–1500 U/L and 8.75 U/L for CAT, 10–400 pg/mL and 2.5 pg/mL for TNF-α, 100–2400 pg/mL and 25 pg/mL for IFN-γ, 12–400 μg/mL and 3 μg/mL for IgG and 1.2–40 μg/mL and 0.3 μg/mL for IgM, respectively. Absorbance was measured at 450 nm using a microplate reader. All samples were analyzed in duplicate, and samples with concentrations outside the lot-specific standard curve range were re-assayed after appropriate dilution. According to the manufacturer’s quality control specifications, the intra-assay and inter-assay coefficients of variation for the ELISA kits were less than 10% and 15%, respectively. The detection ranges and analytical sensitivities of individual kits followed the corresponding lot-specific instructions, and all reported concentrations fell within the respective standard curve ranges.

### 2.5. Quantitative Real-Time PCR

Jejunal and ileal mRNA expression of tight junction and inflammation-related genes was quantified by qRT-PCR (Table 2). RNA was extracted with TRIzol (Invitrogen, Beijing, China), quantified by NanoDrop One (Thermo, Newark, DE, USA), reverse-transcribed (PrimeScript RT, Takara RR047A), and amplified on an ABI 7500 Fast system using TB Green (Takara RR820A). Expression was normalized to *GAPDH* and calculated by 2^−ΔΔCt.

### 2.6. Intestinal Histology and Morphometric Analysis

Jejunal and ileal tissues were fixed (4% paraformaldehyde), paraffin-embedded, sectioned (5 μm), and H&E-stained. Slides were scanned (Pannoramic MIDI, 3DHISTECH Ltd., Budapest, Hungary) and analyzed (CaseViewer, 3DHISTECH Ltd., Budapest, Hungary; Image-Pro Plus 6.0, Media Cybernetics, Inc., Rockville, MD, USA) to measure villus height, crypt depth, and villus-to-crypt ratio.

### 2.7. Bone Length and Mineral Determination

Femur and tibia lengths were measured with a digital caliper. Femur Ca, P, and ash were determined following GB/T 6438 [19]: Ca by flame photometry after hot H_2_SO_4_ digestion, P by molybdenum–antimony colorimetry (700 nm) after H_2_SO_4_–H_2_O_2_ digestion, and ash by dry ashing to constant weight.

### 2.8. Colostrum Routine Composition Analysis

Colostrum was stored at −80 °C, thawed at 4 °C, warmed (~40 °C), and mixed. Fat, protein, lactose, and total solids were measured using an FTIR milk analyzer (MilkoScan FT series, FOSS Analytical A/S, Hillerød, Denmark) in duplicate.

### 2.9. Statistical Analysis

Statistical analyses were performed using SAS 9.4. For each response variable, data were analyzed by one-way ANOVA using the GLM procedure according to Y_ij_ = μ + T_i_ + ε_ij_, where μ is the overall mean, T_i_ is the fixed effect of dietary treatment, and ε_ij_ is the residual error. The sow was the experimental unit for sow and litter outcomes, and the litter was the experimental unit for piglet serum and tissue outcomes (one piglet per litter). Farrowing and weaning endpoints were analyzed separately; therefore, no repeated-measures term was fitted. Residual normality and homogeneity of variance were evaluated before inference, and no material violations were detected. For litter performance, the actual litter size and composition after within-treatment cross-fostering were used in calculating outcomes; litter size and piglet sex were not fitted as separate covariates. Means were compared using Tukey’s HSD test. Results are presented as mean ± SEM; *p* < 0.05 was considered significant and 0.05 ≤ *p* < 0.10 was considered a tendency.

## 3. Results

### 3.1. Effects of Maternal Vitamin D_3_ and 25-OH-D_3_ Supplementation on Sow Reproductive Performance and Piglet Growth

As shown in Table 3, treatment did not affect sow reproductive performance (*p* > 0.05). QV2 increased litter weight on day 14 and litter weight gain from birth to day 14 compared with all other treatments (*p* < 0.05). This early response did not persist to weaning: average piglet weight, litter weight, and litter weight gain from birth to weaning did not differ among treatments (*p* > 0.05).

### 3.2. Effects of Maternal 25-OH-D_3_ Supplementation on Chemical Compositions of Colostrum of Sows

As shown in Table 4, maternal VD_3_ and 25-OH-D_3_ supplementation significantly increased colostrum 25-OH-D_3_ concentration and ALP activity compared with the control group (*p* < 0.01), with QV2 showing the highest 25-OH-D_3_ concentration among all treatments. Colostrum Ca and P concentrations were not affected by treatment (*p* > 0.05).

In colostrum, maternal 25-OH-D_3_ supplementation reduced MDA concentration and enhanced SOD, GSH-Px, and CAT activities compared with the control group (*p* < 0.01), whereas VD_3_ supplementation only increased SOD activity (*p* < 0.01). No significant differences in these indices, except for higher SOD activity, were found between the control and VD_3_ groups (*p* > 0.05).

For cytokines, IL-10 concentration was higher in the VD_3_ and QV3 groups than in the control group (*p* < 0.05), and TNF-α concentration was lower in the QV1 group than in the control group (*p* < 0.05). IL-1β, IL-6, and IFN-γ were not significantly affected by treatment.

### 3.3. Effects of Maternal Vitamin D_3_ and 25-OH-D_3_ Supplementation on Serum 25-OH-D_3_, ALP, Calcium, and Phosphorus in Sows and Piglets

As shown in Table 5, maternal VD_3_ supplementation did not affect sow serum 25-OH-D_3_ at farrowing but significantly increased ALP activity (*p* < 0.01). At weaning, VD_3_ increased serum 25-OH-D_3_ concentration in sows and piglets without altering ALP activity. In contrast, maternal 25-OH-D_3_ supplementation significantly increased serum 25-OH-D_3_ concentration and ALP activity in sows at both farrowing and weaning, as well as in weaning piglets, compared with the control (*p* < 0.01). Serum Ca and P concentrations were not affected by treatment in sows or piglets (*p* > 0.05).

### 3.4. Effects of Maternal Vitamin D_3_ and 25-OH-D_3_ Supplementation on Downstream Vitamin D Metabolites and Vitamin D-Binding Protein in Sows and Piglets

As shown in Table 6, serum concentrations of 1,25-(OH)_2_-D_3_, 24,25-(OH)_2_-D_3_, and DBP did not differ among groups on gestation day 90, indicating comparable pretreatment values. At farrowing, all three 25-OH-D_3_ preparations increased serum 1,25-(OH)_2_-D_3_ compared with the control, with the highest numerical value observed in QV2 (*p* < 0.05). Serum 24,25-(OH)_2_-D_3_ was also increased by additional vitamin D_3_ and by all three 25-OH-D_3_ preparations, with QV2 showing the highest concentration (*p* < 0.05). Serum DBP was unaffected by treatment.

In colostrum, the QV1, QV2, and QV3 treatments increased 1,25-(OH)_2_-D_3_ compared with the control, whereas QV2 produced the highest concentration (*p* < 0.05). Colostrum 24,25-(OH)_2_-D_3_ was higher in all three 25-OH-D_3_ groups than in the control and VD_3_ groups, with the highest values observed in QV2 and QV3 (*p* < 0.05). Colostrum DBP did not differ among treatments.

In weaned piglets, serum and duodenal 1,25-(OH)_2_-D_3_ concentrations were not significantly affected by maternal treatment. In contrast, 24,25-(OH)_2_-D_3_ concentrations in both serum and duodenal tissue were higher in the QV1, QV2, and QV3 groups than in the control and VD_3_ groups, with QV2 and QV3 showing the highest values (*p* < 0.05). DBP concentrations in piglet serum and duodenal tissue were unaffected by treatment.

### 3.5. Effects of Maternal Vitamin D_3_ and 25-OH-D_3_ Supplementation on Serum Antioxidant Status in Sows and Piglets

As shown in Table 7, additional VD_3_ supplementation produced limited effects on serum antioxidant indices, reducing SOD activity in sows at farrowing and increasing GSH-Px activity in piglets at weaning, with no other significant changes. In contrast, all three 25-OH-D_3_ preparations consistently reduced serum MDA concentrations and increased SOD, GSH-Px, and CAT activities in sows at farrowing and weaning and in piglets at weaning (*p* < 0.01).

### 3.6. Effects of Maternal Vitamin D_3_ and 25-OH-D_3_ Supplementation on Serum Inflammatory Cytokines in Sows and Piglets

Table 8 summarizes serum cytokine concentrations. At farrowing, VD_3_ reduced IL-1β and IFN-γ, whereas the three 25-OH-D_3_ preparations produced source-dependent reductions in IL-1β, IL-6, TNF-α, and IFN-γ and increases in IL-10 (*p* < 0.05). At weaning, sow serum IL-1β, IL-6, TNF-α, and IFN-γ differed among treatments, whereas IL-10 was unaffected. The 25-OH-D_3_ preparations generally reduced pro-inflammatory cytokine concentrations, with QV2 and QV3 showing the most consistent reductions. In weaning piglets, IL-10 was unaffected, whereas IL-1β, IL-6, TNF-α, and IFN-γ showed source-dependent treatment responses (*p* < 0.05).

### 3.7. Effects of Maternal Vitamin D_3_ and 25-OH-D_3_ Supplementation on Skeletal Development in Piglets

As shown in Table 9, maternal 25-OH-D_3_ supplementation significantly increased bone Ca content in weaning piglets compared with the control and VD_3_ groups (*p* < 0.01), with the highest value observed in QV2, followed by QV3. No differences were observed in femur or tibia length, bone P content, or bone ash percentage among treatments (*p* > 0.05).

### 3.8. Effects of Maternal Vitamin D_3_ and 25-OH-D_3_ Supplementation on Intestinal Villus Development and Morphology in Piglets

**Figure 1:**
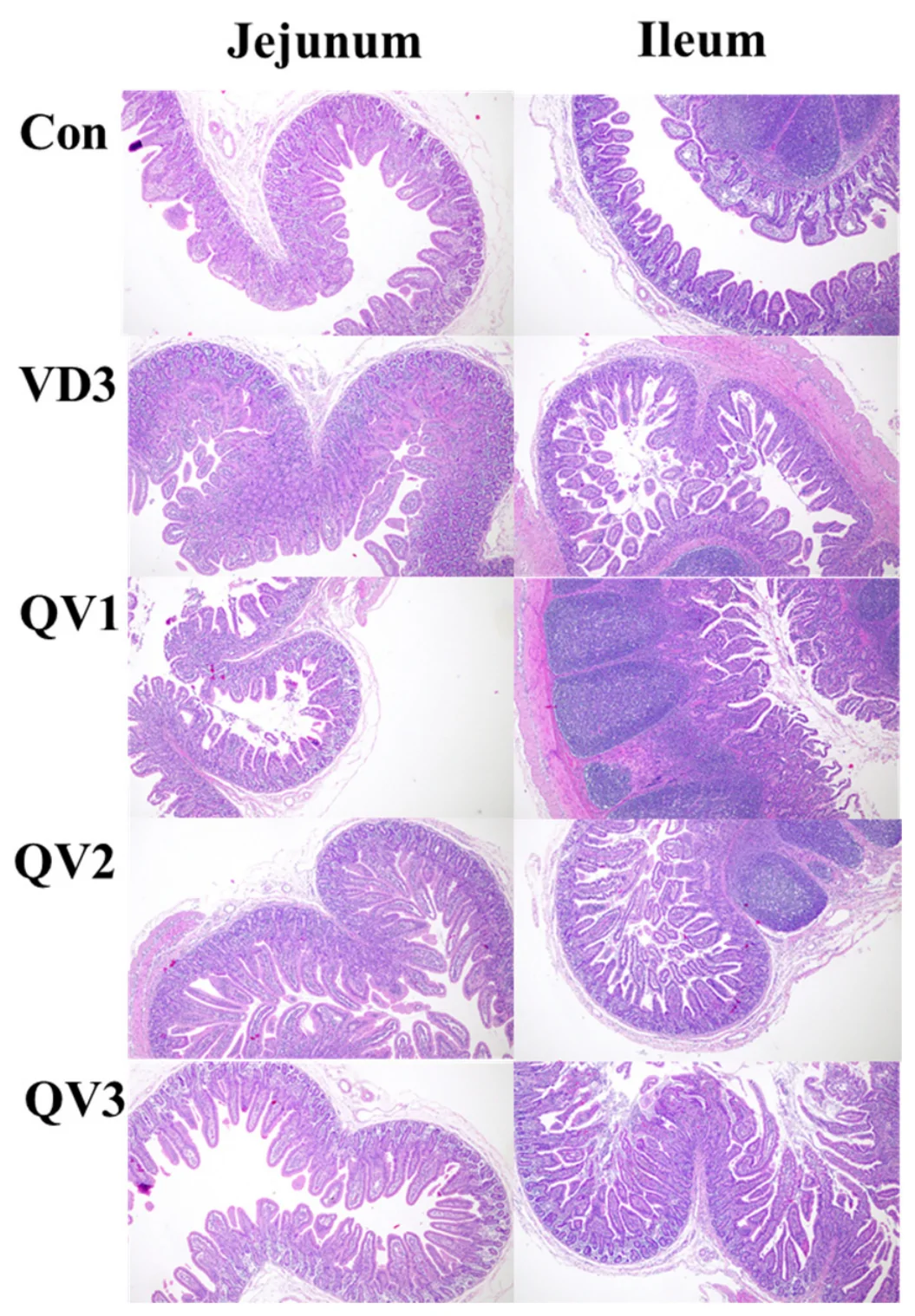
Effects of maternal 25-OH-D_3_ supplementation from day 90 of gestation on jejunal and ileal morphology in weaning piglets. Control: Basal diet; VD_3_ group: The basal diet + 50 μg/kg vitamin D_3_; QV1, QV2, and QV3 groups: The basal diet + 50 μg/kg 25-OH-D_3_ from three different sources.

As shown in Table 10 and Figure 1, jejunal and ileal villus height was significantly increased in the QV2 and QV3 groups compared with the control and VD_3_ groups (*p* < 0.01), whereas QV1 did not differ from the control (*p* > 0.05). In addition, jejunal villus height was also increased in the VD_3_ group compared with the control (*p* < 0.01), while ileal villus height did not differ between these two groups (*p* > 0.05). Consistently, the jejunal villus-to-crypt (V/C) ratio was significantly higher in QV2 and QV3 than in the other treatments (*p* < 0.01).

### 3.9. Effects of Maternal Vitamin D_3_ and 25-OH-D_3_ Supplementation on Gene Expression of Tight Junction Proteins and Inflammatory Cytokines in Piglet Intestines

**Figure 2:**
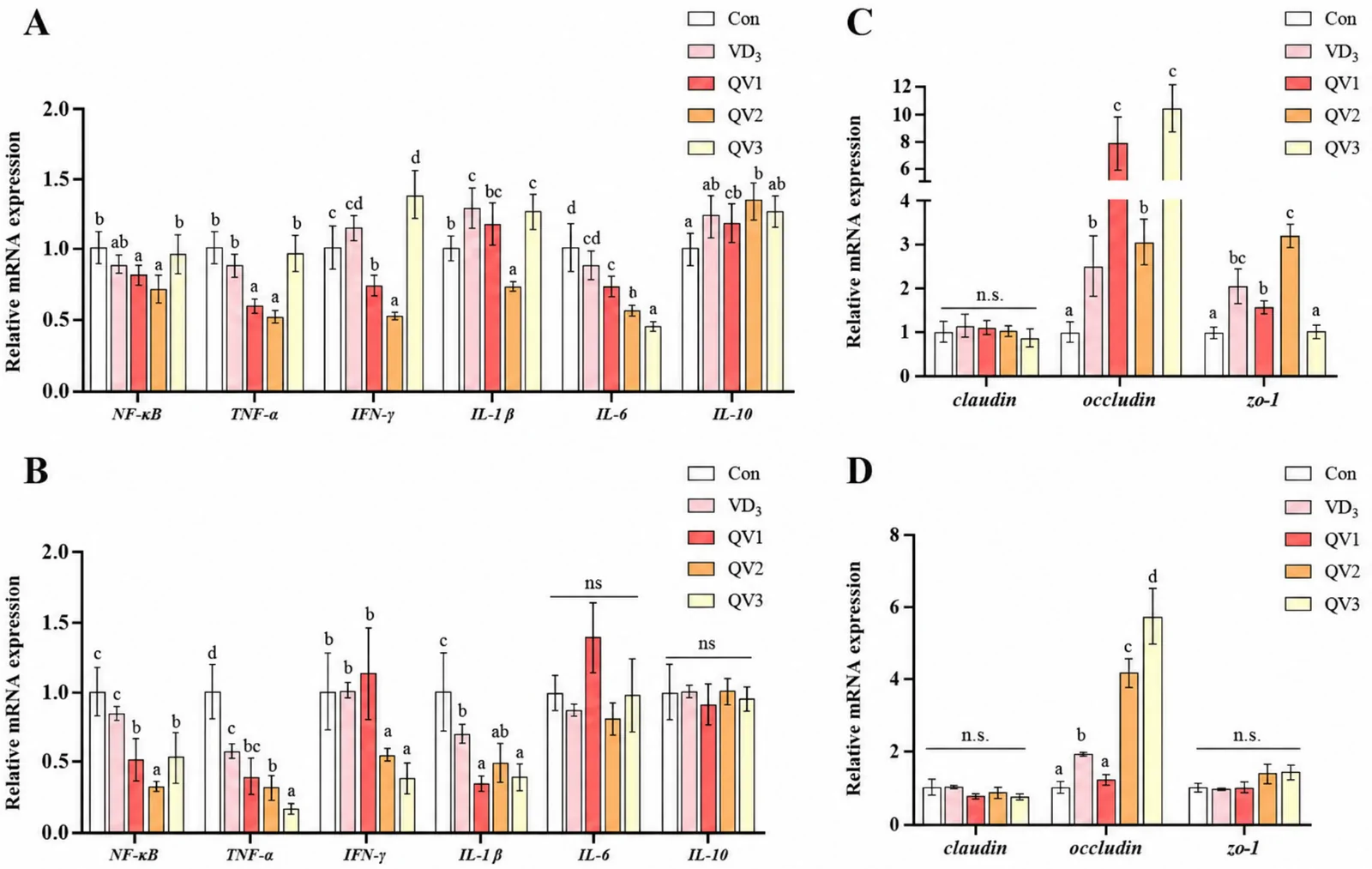
Effects of maternal 25-OH-D_3_ supplementation from day 90 of gestation on gene expression in the jejunum and ileum of weaning piglets (*n* = 6). (**A**) Inflammatory cytokine gene expression in the jejunum; (**B**) Inflammatory cytokine gene expression in the ileum; (**C**) Tight junction protein gene expression in the jejunum; (**D**) Tight junction protein gene expression in the ileum. ^a,b,c,d^ Different superscript letters within a row indicate significant differences among treatments (*p* < 0.05). Control: Basal diet; VD_3_ group: The basal diet + 50 μg/kg vitamin D_3_; QV1, QV2, and QV3 groups: The basal diet + 50 μg/kg 25-OH-D_3_ from three different sources.

As shown in Figure 2A,C, jejunal gene expression responses differed among treatments. Compared with the control, QV2 decreased the mRNA expression of NF-κB, TNF-α, IFN-γ, IL-1β, and IL-6 and increased IL-10 expression (*p* < 0.05). QV1 decreased NF-κB, TNF-α, IFN-γ, and IL-6, increased IL-10, and did not affect IL-1β (*p* < 0.05). QV3 increased IFN-γ and IL-1β, decreased IL-6, and did not affect NF-κB, TNF-α, or IL-10 (*p* < 0.05). VD_3_ increased IL-1β but did not affect NF-κB, TNF-α, IFN-γ, IL-6, or IL-10 relative to the control (*p* < 0.05). Claudin-1 expression was unchanged among treatments (*p* > 0.05), whereas occludin expression was increased in the VD_3_, QV1, QV2, and QV3 groups and ZO-1 expression was increased in the VD_3_, QV1, and QV2 groups (*p* < 0.05).

In the ileum (Figure 2B,D), QV2 decreased NF-κB, TNF-α, IFN-γ, and IL-1β expression compared with the control (*p* < 0.05). QV1 decreased NF-κB, TNF-α, and IL-1β but did not affect IFN-γ, whereas QV3 decreased all four transcripts (*p* < 0.05). VD_3_ decreased TNF-α and IL-1β but did not affect NF-κB or IFN-γ (*p* < 0.05). Ileal IL-6 and IL-10 expression did not differ among treatments (*p* > 0.05). Claudin-1 and ZO-1 expression were also unaffected (*p* > 0.05), whereas occludin expression was increased in the VD_3_ (*p* < 0.05), QV2, and QV3 groups but remained unchanged in QV1.

## 4. Discussion

Maternal 25-OH-D_3_ supplementation increased serum 25-OH-D_3_ concentrations in sows at farrowing and weaning and in piglets at weaning relative to the CON and VD_3_ groups; QV2 also yielded the highest colostrum concentration. However, treatment rankings were not uniform across endpoints: QV2 produced the strongest responses for selected outcomes, whereas QV1 or QV3 was similar or superior for others. The data therefore indicate product-specific responses among the three preparations tested and do not establish that biosynthesized 25-OH-D_3_ is generally superior to chemically synthesized 25-OH-D_3_.

Additional profiling of downstream vitamin D metabolites supported the treatment-related changes in vitamin D status. The absence of differences in 1,25-(OH)_2_-D_3_, 24,25-(OH)_2_-D_3_, and DBP on gestation day 90 confirmed comparable baseline conditions among treatments. At farrowing, maternal 25-OH-D_3_ supplementation increased serum and colostrum concentrations of both 1,25-(OH)_2_-D_3_ and 24,25-(OH)_2_-D_3_, with QV2 generally showing the strongest response. These findings are consistent with the capacity of porcine tissues to convert 25-OH-D_3_ through both 1α-hydroxylation and 24-hydroxylation pathways [20,21]. Similarly, Thayer et al. reported increased serum 24,25-(OH)_2_-D_3_ in progeny from 25-OH-D_3_-supplemented sows, although no significant effect was detected in colostrum or milk [22]. Differences in product formulation, sampling conditions, and analytical methods may account for the different colostrum responses. In weaned piglets, the increase in serum and duodenal 24,25-(OH)_2_-D_3_ without a corresponding increase in 1,25-(OH)_2_-D_3_ may reflect increased vitamin D turnover under homeostatic regulation. DBP remained unchanged across maternal and offspring samples, indicating that the changes in total vitamin D metabolites were not accompanied by detectable alterations in carrier abundance [23].

Despite the increase in 25-OH-D_3_ status, serum and colostrum calcium and phosphorus were unchanged, consistent with tight homeostatic regulation [24]. Piglet femoral calcium concentration increased by 6–14% with the 25-OH-D_3_ treatments, with the largest value in QV2, but bone length, phosphorus concentration, and ash were unaffected. Because total ALP rather than a bone-specific ALP isoenzyme was measured, the higher ALP activity should be interpreted only as supportive evidence of altered mineral or bone metabolic activity, not as direct proof of enhanced osteoblast activity, mineral deposition, skeletal development, or bone strength [25]. These observations are broadly consistent with reports that maternal 25-OH-D_3_ affects vitamin D status and selected indices of skeletal mineralization [11,26,27].

Maternal 25-OH-D_3_ supplementation did not affect litter size or birth weight. The significant growth response was confined to the first 14 days of life: QV2 increased litter weight on day 14 and litter weight gain from birth to day 14 by 9.8% relative to CON, whereas average piglet weight on day 14 showed only a tendency (*p* = 0.09). One potential contributing factor is that, at approximately 21 days of lactation, some clinically smaller piglets were cross-fostered by farm personnel to other sows within the same dietary treatment. Although restricting transfers to the same treatment prevented direct cross-treatment contamination, late-lactation cross-fostering altered the composition of individual litters and may have increased variation in litter and piglet. weights at weaning, thereby attenuating the apparent persistence of the day-14 response. Litter weight, average piglet weight, and litter weight gain to weaning did not differ among treatments. Previous studies have reported variable growth responses to maternal 25-OH-D_3_ supplementation [14,28,29]. The source-dependent responses observed here may relate to product-specific composition or formulation. Regulatory assessments likewise treat manufacturing process, composition, and stability as product-specific attributes [30]. Because epimer/isomer profiles, carrier composition, feed recovery, stability, and pharmacokinetics were not measured, the present study cannot identify the mechanism responsible for differences among QV1, QV2, and QV3.

Maternal 25-OH-D_3_ supplementation altered the overall redox profile in sows, colostrum, and piglets, as reflected by coordinated changes in MDA, SOD, GSH-Px, and CAT rather than by any single antioxidant enzyme; however, the magnitude and direction of individual responses were not uniform across sources or sampling points. This combined pattern of lipid peroxidation and antioxidant defense markers is compatible with improved redox balance in selected matrices. Oxidative stress is common during reproduction and has been associated with placental dysfunction and impaired offspring development [31,32,33]. In weaned piglets, 118 µg/kg 25-OH-D_3_ and doses of 50 or 75 µg/kg have also been associated with combined changes in antioxidant indices [34,35]. Direct ROS measurements and tissue oxidative damage endpoints were not assessed in the present study. Because the intervention began on day 90 of gestation and the basal diet met NRC [17] requirements, unchanged litter size and birth weight were not unexpected. These biochemical changes should therefore be interpreted as marker responses rather than evidence of improved reproductive performance, and the safety and long-term effects of supranutritional supplementation require further evaluation.

Maternal 25-OH-D_3_ supplementation produced source-dependent changes in systemic immune markers. In sow serum, the three 25-OH-D_3_ preparations generally reduced IL-1β, IL-6, TNF-α, and IFN-γ at farrowing and/or weaning, whereas increases in IL-10 were mainly observed at farrowing. In weaned piglets, QV2 and QV3 produced the most consistent reductions in circulating pro-inflammatory cytokines. These changes may be physiologically relevant because weaning is accompanied by increased intestinal expression of IL-1β, IL-6, and TNF-α in piglets [36]. Madsen et al. similarly showed that improving 25-OH-D_3_ status in piglets was associated with altered immune responses and potentially greater robustness during an Escherichia coli challenge, although their study did not demonstrate the same cytokine pattern observed here [37]. Mechanistically, 1,25-(OH)_2_-D_3_–VDR signaling can attenuate Toll-like receptor-mediated inflammation by limiting NF-κB-associated signaling and strengthening negative feedback regulation [38]. Intestinal epithelial VDR signaling has also been shown to reduce mucosal inflammation and epithelial injury in experimental models [39,40]. Collectively, the circulating and intestinal results support source-dependent immunomodulation by maternal 25-OH-D_3_ supplementation, although they should not be interpreted as direct evidence of reduced histological inflammation.

Maternal 25-OH-D_3_ supplementation also altered selected circulating and intestinal inflammatory markers. In piglet intestines, QV2 was associated with lower mRNA abundance of several inflammation-related genes, whereas QV1 and QV3 produced more selective transcriptional changes. These patterns are consistent with altered mucosal immune signaling [41,42,43] but do not directly demonstrate inhibition of intestinal inflammation because protein abundance, pathway activation, immune cell infiltration, and histopathological inflammation were not measured. The immune marker changes may have accompanied the early litter growth response, but the present design does not establish a causal relationship, and the absence of significant effects on weaning weight or total litter gain to weaning warrants cautious interpretation. Mechanistic studies are needed to determine whether differences in absorption, metabolism, or tissue signaling explain the source-dependent responses.

QV2 and QV3 increased jejunal and ileal villus height, and selected 25-OH-D_3_ treatments upregulated occludin and ZO-1 mRNA in a segment- and source-dependent manner. Villus architecture and tight-junction-related transcripts are relevant to absorptive and barrier biology [44,45], but intestinal permeability, nutrient absorption, and tight-junction protein abundance were not measured. Accordingly, these outcomes support changes in intestinal morphology and barrier-related gene expression but do not constitute direct evidence of enhanced intestinal barrier integrity or more efficient nutrient absorption.

### Study Limitations

This study was conducted on one farm using one genotype and one supplemental dose and compared only three commercial preparations. The 25-OH-D_3_ content of each formulated premix was verified by HPLC; however, the chemical purity of the isolated active ingredients, epimer/isomer profiles, and pharmacokinetic characteristics were not independently determined. Therefore, the observed differences should be interpreted as product-specific responses among the preparations tested rather than as general differences between chemical and biosynthetic production routes. Vitamin D pharmacokinetics were not characterized by serial sampling. The biochemical and tissue subset comprised six independent sows or litters per treatment, cross-fostering occurred within treatment, and piglet sex was not controlled at terminal sampling. Direct ROS and tissue oxidative damage endpoints, inflammatory protein abundance, pathway activation, immune cell infiltration, histopathological inflammation, intestinal permeability, nutrient absorption, tight-junction proteins, bone strength, long-term reproductive outcomes, and economic return were not evaluated. These limitations restrict mechanistic interpretation and generalization of source-dependent effects.

## 5. Conclusions

Maternal supplementation with 25-OH-D_3_ from late gestation to weaning increased 25-OH-D_3_ status in sows and piglets and altered selected redox, inflammatory, femoral calcium, intestinal morphology, and barrier-related gene expression endpoints. QV2 increased litter weight gain from birth to day 14 and produced the largest femoral calcium response, but litter and average piglet weights at weaning were unchanged. QV2 and QV3 increased jejunal and ileal villus height, and selected treatments upregulated occludin and ZO-1 mRNA; these findings describe intestinal morphology and barrier-related gene expression but do not constitute direct evidence of enhanced intestinal barrier integrity. Under the conditions of the present study, QV2 showed the strongest responses for selected endpoints among the three preparations tested. Because the products were incompletely characterized and were evaluated at one dose under one production setting, the findings do not establish general superiority of biosynthesized products. Additional product characterization, dose–response studies, serial vitamin D metabolite and binding protein measurements, independent validation, long-term production studies, and economic evaluation are required before a source-specific practical recommendation can be made.

## Figures and Tables

**Table 1:** Ingredient and nutrient composition of the basal diets (% as fed).

	Gestation	Lactation
Ingredient (%)		
Corn	46.49	58.99
Soybean meal	5.00	11.00
Wheat bran	22.00	8.00
Wheat feed flour	5.00	8.00
Expanded soybean	-	10.00
Rice bran	10.00	-
Rice bran meal	8.00	-
Salt	0.50	-
Soybean oil	0.50	1.00
Limestone	1.50	-
Premix ^1^	1.00	1.00
Fish meal	0.00	2.00
Modified starch	0.01	0.01
Total	100.00	100.00
Nutrients		
Crude protein (%)	12.18	16.00
Digestible energy (kcal/kg)	3030	3334
Metabolizable energy (kcal/kg)	2897	3157
Net energy (kcal/kg)	2253	2455
Dry matter (%)	87.16	80.40
Crude fat (%)	5.00	5.40
Crude fiber (%)	4.58	3.04
Acid detergent fiber (%)	5.95	3.83
Neutral detergent fiber (%)	16.19	10.42
Crude ash (%)	6.03	5.68
Calcium (%)	0.67	0.88
Total phosphorus (%)	0.68	0.59
Available phosphorus (%)	0.26	0.38
Salt (%)	0.58	0.51
Lysine (%)	0.65	1.03
Total Fiber (%)	19.94	14.06
Soluble Fiber (%)	3.09	1.66
Insoluble Fiber (%)	16.75	12.26
Vitamin D_3_ (μg/kg)	25	25

^1^ Composition of 1 kg premix: Vitamin A, 130–175 kIU; vitamin D_3_, 2500 μg (100 kIU); Vitamin E, 500 IU; Vitamin K3, ≥45 mg; Vitamin B1, ≥50 mg; Vitamin B2, ≥150 mg; Vitamin B6, ≥100 mg; Vitamin B12, ≥0.5 mg; Niacin, 650 mg; Pantothenic acid, ≥450 mg; Folic acid, ≥80 mg; Biotin, ≥10 mg; Fe, 2.4~18 g; Cu, 0.2~0.62 g; Zn, 1~2.5 g; Mn, 0.5~2 g; I, 10~50 mg; Se, 5~12.5 mg; Calcium, 80 g; Phosphorus, 60 g; Choline chloride, ≥10 mg; Moisture, ≤10%.

**Table 2:** Primers (pig) used for qPCR.

Genes	Forward (5′–3′)	Reverse (5′–3′)	Product Size (bp)
*GAPDH*	CGTCCCTGAGACACGATGGT	CCCGATGCGGCCAAAT	54
*IL-10*	CGGCGCTGTCATCAATTTCTG	CCCCTCTCTTGGAGCTTGCTA	196
*IL-1β*	CAACGTGCAGTCTATGGAGT	GAGGTGCTGATGTACCAGTTG	180
*IL-6*	TACTGGCAGAAAACAACCTG	GTACTAATCTGCACAGCCTC	177
*TNF-α*	TCCAATGGCAGAGTGGGATG	AGCTGGTTGTCTTTCAGCTTCAC	81
*IFN-γ*	GGATTTGCCCTGACCCTACT	TCTCTGTGCTGACATCGCTC	199
*NF-* *κ* *B*	TTTCACTTGTCCCGCTCTCC	CGCCTTTGGAATTGCCTTGA	178
*Claudin*	CTGTTTGCCCATGTTTGGCT	TCCAGCCAATCTTCTCGTCA	167
*Occludin*	TATGAGACAGACTACACAACTGGCGGCGAGTCC	ATCATAGTCTCCAACCATCTTCTTGATGTG	215
*Z* *O* *-* *1*	AGGCGATGTTGTATTGAAGATAAATG	TTTTTGCATCCGTCAATGACA	217

**Table 3:** Effects of maternal 25-OH-D_3_ supplementation from day 90 of gestation on reproductive performance of sows and growth performance of piglets (*n* = 20).

Item	Control	VD_3_	QV1	QV2	QV3	SEM	*p*-Value
Sow performance							
Litter size	13.68	14.11	13.91	14.22	13.90	0.24	0.96
Number of born alive	13.14	13.53	13.45	13.70	13.30	0.27	0.97
Number of stillbirths	0.54	0.58	0.45	0.52	0.60	0.07	0.98
Number of healthy piglets	12.73	12.90	12.95	13.26	12.70	0.29	0.97
Number of weak piglets	0.41	0.63	0.50	0.43	0.60	0.07	0.80
Backfat loss (mm)	2.52	2.80	2.83	2.57	2.88	0.06	0.19
Average birth weight (kg)	1.41	1.34	1.39	1.46	1.41	0.02	0.21
Litter weight at birth (kg)	18.43	18.30	18.55	19.85	18.65	0.36	0.73
Piglet performance							
Litter weight on day 14 (kg)	47.25 ^a^	47.59 ^a^	46.15 ^a^	51.58 ^b^	47.15 ^a^	0.61	0.04
Litter weight gain from birth to day 14 (kg)	29.10 ^a^	29.29 ^a^	27.59 ^a^	31.94 ^b^	28.50 ^a^	0.33	<0.01
Average weight on day 14 (kg)	3.56	3.67	3.75	3.84	3.77	0.03	0.09
Litter weight at weaning (kg)	93.21	91.83	92.14	98.27	92.30	1.07	0.25
Litter weight gain from birth to weaning (kg)	75.65	73.46	73.38	78.63	73.42	1.00	0.36
Average weight at weaning (kg)	7.20	7.23	7.34	7.53	7.39	0.05	0.22

^a,b^ Different superscript letters within a row indicate significant differences among treatments (*p* < 0.05). Control: Basal diet; VD_3_ group: The basal diet + 50 μg/kg Vitamin D_3_; QV1, QV2, and QV3 groups: The basal diet + 50 μg/kg 25-OH-D_3_ from three different sources.

**Table 4:** Effects of maternal 25-OH-D_3_ supplementation from day 90 of gestation on chemical compositions of colostrum of sows (*n* = 6).

Item	Control	VD_3_	QV1	QV2	QV3	SEM	*p*-Value
Protein (%)	12.62	12.98	12.95	12.72	13.05	0.17	0.49
Fat (%)	2.51	2.35	2.53	2.67	2.86	0.04	0.69
Lactose (g/L)	50.54	46.95	47.54	50.01	48.16	0.78	0.55
Total solids (%)	22.14	21.90	22.41	21.57	21.84	0.23	0.90
IgG (mg/mL)	40.49	40.19	39.67	41.39	40.73	0.32	0.94
IgM (mg/mL)	3.13	3.19	3.27	3.32	3.05	0.04	0.23
25-OH-D_3_ (μg/L)	12.73 ^a^	13.42 ^b^	14.21 ^c^	14.91 ^d^	13.80 ^bc^	0.16	<0.01
Ca (mmol/L)	4.89	4.95	4.91	4.84	4.80	0.07	0.99
P (mmol/L)	1.29	1.19	1.27	1.25	1.30	0.03	0.22
ALP (U/L)	70.54 ^a^	95.43 ^c^	78.52 ^b^	84.16 ^b^	81.95 ^b^	1.97	<0.01
MDA (nmol/L)	8.73 ^b^	9.42 ^b^	7.16 ^a^	7.11 ^a^	6.62 ^a^	0.25	<0.01
SOD (U/L)	1357.37 ^a^	1479.65 ^b^	1534.27 ^b^	1549.61 ^b^	1554.34 ^b^	21.36	<0.01
GSH-Px (IU/L)	120.00 ^ab^	117.56 ^a^	144.78 ^c^	145.96 ^c^	132.56 ^bc^	3.12	<0.01
CAT (U/L)	731.07 ^a^	753.83 ^a^	849.91 ^b^	909.37 ^b^	918.46 ^b^	20.02	<0.01
IL-10 (ng/L)	139.79 ^a^	145.16 ^b^	144.33 ^ab^	143.18 ^ab^	147.99 ^b^	0.87	0.03
IL-1β (ng/L)	41.24	41.92	43.40	41.16	39.93	0.45	0.16
IL-6 (ng/L)	663.11	699.44	658.80	686.03	654.36	8.77	0.44
TNF-α (ng/L)	244.96 ^b^	225.75 ^ab^	203.78 ^a^	238.03 ^b^	233.35 ^b^	4.65	0.04
IFN-γ (ng/L)	2187.92	2150.85	2219.69	2102.60	2132.21	14.99	0.09

Abbreviations: IgG, immunoglobulin G; IgM, immunoglobulin M; 25-OH-D_3_: 25-Hydroxyvitamin D_3_; ALP: Alkaline phosphatase; MDA: Malondialdehyde; SOD: Superoxide dismutase; GSH-Px: Glutathione peroxidase; CAT: Catalase; IL-10: Interleukin-10; IL-1β: Interleukin-1 beta; IL-6: Interleukin-6; TNF-α: Tumor necrosis factor-alpha; IFN-γ: Interferon-gamma. Ca: calcium; P: phosphorus. Control: Basal diet; VD_3_ group: The basal diet + 50 μg/kg Vitamin D_3_; QV1, QV2, and QV3 groups: The basal diet + 50 μg/kg 25-OH-D_3_ from three different sources. ^a,b,c,d^ Different superscript letters within a row indicate significant differences among treatments (*p* < 0.05).

**Table 5:** Effects of maternal 25-OH-D_3_ supplementation from day 90 of gestation on serum statuses of vitamin D, Ca, and P in sows and piglets.

Item	Control	VD_3_	QV1	QV2	QV3	SEM	*p*-Value
Sows at farrowing (*n* = 6)							
25-OH-D_3_ (μg/L)	14.83 ^a^	14.71 ^a^	17.32 ^b^	17.26 ^b^	17.10 ^b^	0.26	<0.01
ALP (U/L)	67.24 ^a^	76.35 ^b^	90.53 ^c^	91.98 ^c^	89.52 ^c^	2.33	<0.01
Ca (mmol/L)	2.79	3.12	2.94	3.03	3.21	0.06	0.286
P (mmol/L)	1.22	1.31	1.28	1.25	1.21	0.03	0.711
Sows at weaning (*n* = 6)							
25-OH-D_3_ (μg/L)	12.49 ^a^	13.22 ^b^	14.84 ^c^	14.36 ^c^	14.85 ^c^	0.21	<0.01
ALP (U/L)	77.54 ^a^	78.30 ^a^	99.73 ^c^	90.00 ^b^	100.21 ^c^	2.29	<0.01
Ca (mmol/L)	3.03	3.00	3.12	3.15	3.06	0.03	0.871
P (mmol/L)	1.29	1.34	1.42	1.39	1.45	0.02	0.106
Piglets at weaning (*n* = 6)							
25-OH-D_3_ (μg/L)	14.22 ^a^	15.46 ^b^	18.07 ^c^	18.94 ^c^	17.80 ^d^	0.34	<0.01
ALP (U/L)	80.81 ^a^	87.05 ^a^	111.69 ^c^	98.04 ^b^	96.32 ^b^	2.26	<0.01
Ca (mmol/L)	3.10	3.12	3.18	3.27	3.15	0.04	0.775
P (mmol/L)	1.36	1.45	1.43	1.49	1.44	0.02	0.303

Abbreviations: 25-OH-D_3_: 25-Hydroxyvitamin D_3_; ALP: Alkaline phosphatase; Ca: Blood calcium; P: Blood phosphorus. Control: Basal diet; VD_3_ group: The basal diet + 50 μg/kg Vitamin D_3_; QV1, QV2, and QV3 groups: The basal diet + 50 μg/kg 25-OH-D_3_ from three different sources. ^a,b,c,d^ Different superscript letters within a row indicate significant differences among treatments (*p* < 0.05).

**Table 6:** Effects of maternal vitamin D_3_ and 25-OH-D_3_ supplementation on downstream vitamin D metabolites and vitamin D-binding protein in sows and weaned piglets.

Item	Control	VD_3_	QV1	QV2	QV3	SEM	*p*-Value
Sow serum at gestation day 90 (*n* = 6)							
1,25-(OH)_2_-D_3_ (pg/mL)	108.67	106.93	99.19	100.25	103.00	4.82	0.580
24,25-(OH)_2_-D_3_ (ng/mL)	1.99	2.19	2.04	2.09	2.02	0.09	0.608
DBP (μg/mL)	346.95	308.13	326.81	339.00	329.73	15.98	0.514
Sow serum at farrowing (*n* = 6)							
1,25-(OH)_2_-D_3_ (pg/mL)	103.47 ^c^	109.92 ^bc^	120.01 ^ab^	129.78 ^a^	124.09 ^ab^	3.78	<0.01
24,25-(OH)_2_-D_3_ (ng/mL)	1.16 ^d^	1.35 ^c^	1.63 ^b^	2.00 ^a^	1.80 ^b^	0.05	<0.01
DBP (μg/mL)	648.91	679.49	590.94	653.08	622.97	29.52	0.300
Sow colostrum (*n* = 6)							
1,25-(OH)_2_-D_3_ (pg/mL)	150.72 ^c^	159.95 ^bc^	179.01 ^ab^	186.93 ^a^	181.55 ^ab^	5.69	<0.01
24,25-(OH)_2_-D_3_ (ng/mL)	0.71 ^c^	0.76 ^c^	1.02 ^b^	1.19 ^a^	1.15 ^a^	0.03	<0.01
DBP (μg/mL)	529.97	517.95	491.39	500.61	498.18	15.20	0.383
Weaned piglet serum (*n* = 6)							
1,25-(OH)_2_-D_3_ (pg/mL)	88.54	99.80	101.85	103.65	96.64	4.50	0.172
24,25-(OH)_2_-D_3_ (ng/mL)	1.01 ^c^	1.07 ^c^	1.34 ^b^	1.63 ^a^	1.56 ^a^	0.05	<0.01
DBP (μg/mL)	363.77	397.48	377.59	379.89	423.10	22.78	0.425
Piglet duodenal tissue (*n* = 6)							
1,25-(OH)_2_-D_3_ (pg/g)	105.04	119.46	120.86	122.29	111.75	5.27	0.139
24,25-(OH)_2_-D_3_ (ng/g)	1.75 ^c^	1.86 ^c^	2.20 ^b^	2.62 ^a^	2.67 ^a^	0.06	<0.001
DBP (μg/g)	420.56	416.96	429.60	381.09	403.03	22.51	0.596

Abbreviations: 1,25-(OH)_2_-D_3_: 1,25-Dihydroxyvitamin D_3_; 24,25-(OH)_2_-D_3_: 24,25-Dihydroxyvitamin D_3_; DBP: Vitamin D-binding protein. Control: Basal diet; VD_3_ group: The basal diet + 50 μg/kg Vitamin D_3_; QV1, QV2, and QV3 groups: The basal diet + 50 μg/kg 25-OH-D_3_ from three different sources. ^a,b,c,d^ Different superscript letters within a row indicate significant differences among treatments (*p* < 0.05).

**Table 7:** Effects of maternal 25-OH-D_3_ supplementation from day 90 of gestation on serum antioxidant status in sows and piglets.

Item	Control	VD_3_	QV1	QV2	QV3	SEM	*p*-Value
Sows at farrowing (*n* = 6)							
MDA (nmol/L)	10.46 ^c^	10.29 ^c^	8.09 ^ab^	8.33 ^b^	7.49 ^a^	0.26	<0.01
SOD (U/L)	1004.11 ^b^	852.74 ^a^	1237.22 ^c^	1313.50 ^c^	1336.74 ^c^	41.66	<0.01
GSH-Px (IU/L)	124.83 ^a^	127.12 ^a^	203.19 ^b^	213.54 ^bc^	218.24 ^c^	8.76	<0.01
CAT (U/L)	647.67 ^a^	677.81 ^a^	854.22 ^b^	884.26 ^b^	920.50 ^b^	24.97	<0.01
Sows at weaning (*n* = 6)							
MDA (nmol/L)	9.13 ^b^	8.96 ^b^	6.26 ^a^	5.85 ^a^	5.98 ^a^	0.32	<0.01
SOD (U/L)	1007.60 ^a^	1012.91 ^a^	1347.12 ^b^	1406.83 ^b^	1391.33 ^b^	39.60	<0.01
GSH-Px (IU/L)	106.97 ^a^	112.29 ^a^	170.94 ^b^	182.43 ^b^	166.57 ^b^	6.31	<0.01
CAT (U/L)	545.73 ^a^	627.38 ^a^	705.33 ^b^	850.42 ^b^	746.48 ^b^	23.35	<0.01
Piglets at weaning (*n* = 6)							
MDA (nmol/L)	7.36 ^b^	7.44 ^b^	6.49 ^a^	6.39 ^a^	6.19 ^a^	0.13	<0.01
SOD (U/L)	1373.18 ^a^	1358.45 ^a^	1569.23 ^b^	1650.50 ^b^	1613.54 ^b^	27.04	<0.01
GSH-Px (IU/L)	111.04 ^a^	124.47 ^b^	169.50 ^c^	172.60 ^c^	176.01 ^c^	5.39	<0.01
CAT (U/L)	521.88 ^a^	538.65 ^a^	709.45 ^b^	709.40 ^b^	769.53 ^b^	20.63	<0.01

Abbreviations: MDA: Malondialdehyde; SOD: Superoxide dismutase; GSH-Px: Glutathione peroxidase; CAT: Catalase. Control: Basal diet; VD_3_ group: The basal diet + 50 μg/kg Vitamin D_3_; QV1, QV2, and QV3 groups: The basal diet + 50 μg/kg 25-OH-D_3_ from three different sources. ^a,b,c^ Different superscript letters within a row indicate significant differences among treatments (*p* < 0.05).

**Table 8:** Effects of maternal 25-OH-D_3_ supplementation from day 90 of gestation on serum levels of inflammatory cytokines in sows and piglets.

Item	Control	VD_3_	QV1	QV2	QV3	SEM	*p*-value
Sows at farrowing (*n* = 6)							
IL-10 (ng/L)	141.87 ^a^	150.31 ^ab^	165.05 ^c^	151.20 ^ab^	157.23 ^bc^	2.23	<0.01
IL-1β (ng/L)	41.40 ^d^	36.95 ^c^	28.11 ^a^	28.92 ^a^	33.59 ^b^	1.08	<0.01
IL-6 (ng/L)	574.49 ^b^	548.42 ^b^	362.09 ^a^	397.88 ^a^	371.78 ^a^	19.87	<0.01
TNF-α (ng/L)	279.25 ^b^	288.09 ^b^	260.34 ^b^	194.14 ^a^	180.58 ^a^	12.55	<0.01
IFN-γ (ng/L)	1880.05 ^d^	1771.85 ^c^	1473.01 ^a^	1617.76 ^b^	1717.07 ^bc^	31.90	<0.01
Sows at weaning (*n* = 6)							
IL-10 (ng/L)	156.47	166.17	163.06	162.78	164.11	2.32	0.774
IL-1β (ng/L)	41.51 ^c^	42.56 ^c^	29.43 ^a^	30.42 ^a^	38.27 ^b^	1.18	<0.01
IL-6 (ng/L)	669.66 ^c^	642.60 ^c^	448.55 ^a^	474.87 ^a^	550.15 ^b^	18.99	<0.01
TNF-α (ng/L)	242.71 ^c^	218.73 ^c^	182.69 ^b^	134.45 ^a^	138.12 ^a^	9.26	<0.01
IFN-γ (ng/L)	1697.13 ^b^	1856.16 ^c^	1673.31 ^b^	1381.40 ^a^	1363.49 ^a^	40.73	<0.01
Piglets at weaning (*n* = 6)							
IL-10 (ng/L)	150.52	151.49	147.02	154.55	159.47	1.60	0.538
IL-1β (ng/L)	31.42 ^c^	36.32 ^d^	29.55 ^c^	23.47 ^a^	26.61 ^b^	0.85	<0.01
IL-6 (ng/L)	582.99 ^c^	549.44 ^c^	416.95 ^b^	331.77 ^a^	319.62 ^a^	21.56	<0.01
TNF-α (ng/L)	289.94 ^b^	243.88 ^a^	218.32 ^a^	217.11 ^a^	239.76 ^a^	6.11	<0.01
IFN-γ (ng/L)	1974.62 ^c^	1802.18 ^b^	1786.49 ^b^	1392.37 ^a^	1366.73 ^a^	46.13	<0.01

Abbreviations: IL-10: Interleukin-10; IL-1β: Interleukin-1 beta; IL-6: Interleukin-6; TNF-α: Tumor necrosis Factor-alpha; IFN-γ: Interferon-gamma. Control: Basal diet; VD_3_ group: The basal diet + 50 μg/kg Vitamin D_3_; QV1, QV2, and QV3 groups: The basal diet + 50 μg/kg 25-OH-D_3_ from three different sources. ^a,b,c,d^ Different superscript letters within a row indicate significant differences among treatments (*p* < 0.05).

**Table 9:** Effects of maternal 25-OH-D_3_ supplementation from day 90 of gestation on bone length and Ca and P contents in weaning piglets (*n* = 6).

Item	Control	VD_3_	QV1	QV2	QV3	SEM	*p*-Value
Average piglet weight (kg)	8.37	8.50	8.43	8.33	8.47	0.07	0.95
Femur length (cm)	8.22	8.42	8.43	8.58	8.33	0.11	0.91
Tibia length (cm)	7.47	8.03	7.78	7.85	7.85	0.10	0.57
Ca (g/kg)	160.89 ^a^	157.90 ^a^	171.05 ^b^	184.04 ^c^	176.43 ^bc^	2.17	<0.01
P (%)	5.84	5.69	5.95	5.90	5.76	0.04	0.19
Ash (%)	39.03	40.90	42.63	42.20	41.09	0.51	0.21

Abbreviations: Ca: Bone calcium; P: Bone phosphorus. Control: Basal diet; VD_3_ group: The basal diet + 50 μg/kg Vitamin D_3_; QV1, QV2, and QV3 groups: The basal diet + 50 μg/kg 25-OH-D_3_ from three different sources. ^a,b,c^ Different superscript letters within a row indicate significant differences among treatments (*p* < 0.05).

**Table 10:** Effects of maternal 25-OH-D_3_ supplementation from day 90 of gestation on intestinal villus characters in weaning piglets (*n* = 6).

Item	Control	VD_3_	QV1	QV2	QV3	SEM	*p*-Value
Jejunum							
Villus height (μm)	274.41 ^a^	383.15 ^b^	344.39 ^ab^	521.74 ^c^	539.22 ^c^	29.39	<0.01
Crypt depth (μm)	244.17	319.84	265.42	207.93	218.22	19.68	0.437
V/C ratio	1.12 ^a^	1.32 ^a^	1.37 ^a^	2.49 ^b^	2.59 ^b^	0.19	<0.01
Ileum							
Villus height (μm)	289.05 ^ab^	221.71 ^a^	364.31 ^bc^	454.57 ^c^	424.22 ^c^	26.26	<0.01
Crypt depth (μm)	214.85	134.57	200.03	183.12	191.26	15.25	0.588
V/C ratio	1.42	1.68	1.84	2.73	2.32	0.17	0.07

Abbreviations: V/C ratio: Villus height/Crypt depth ratio; Control: Basal diet; VD_3_ group: The basal diet + 50 μg/kg Vitamin D_3_; QV1, QV2, and QV3 groups: The basal diet + 50 μg/kg 25-OH-D_3_ from three different sources. ^a,b,c^ Different superscript letters within a row indicate significant differences among treatments (*p* < 0.05).

## Data Availability

The data presented in this study are available on request from the corresponding author.

## Abbreviations

The following abbreviations are used in this manuscript:

ALP	alkaline phosphatase
CAT	catalase
DBP	Vitamin D-binding protein
GSH-Px	glutathione peroxidase
IFN-γ	interferon-gamma
IgG	Immunoglobulin G
IgM	Immunoglobulin M
IL	interleukin
MDA	malondialdehyde
NF-κB	Nuclear factor kappa B
qRT-PCR	quantitative real-time PCR
SOD	superoxide dismutase
TNF-α	tumor necrosis factor-alpha
V/C ratio	villus height-to-crypt depth ratio
VDR	Vitamin D receptor
ZO-1	Zonula occludens-1
1,25-(OH)_2_-D_3_	1,25-Dihydroxyvitamin D_3_
24,25-(OH)_2_-D_3_	24,25-Dihydroxyvitamin D_3_
25-OH-D_3_	25-hydroxyvitamin D_3_
